# Maximizing the optical network capacity

**DOI:** 10.1098/rsta.2014.0440

**Published:** 2016-03-06

**Authors:** Polina Bayvel, Robert Maher, Tianhua Xu, Gabriele Liga, Nikita A. Shevchenko, Domaniç Lavery, Alex Alvarado, Robert I. Killey

**Affiliations:** Optical Networks Group, University College London, Torrington Place, London WC1E 7JE, UK

**Keywords:** optical fibre communications, optical nonlinearities, Kerr effect, channel modelling, signal processing, optical networks

## Abstract

Most of the digital data transmitted are carried by optical fibres, forming the great part of the national and international communication infrastructure. The information-carrying capacity of these networks has increased vastly over the past decades through the introduction of wavelength division multiplexing, advanced modulation formats, digital signal processing and improved optical fibre and amplifier technology. These developments sparked the communication revolution and the growth of the Internet, and have created an illusion of infinite capacity being available. But as the volume of data continues to increase, is there a limit to the capacity of an optical fibre communication channel? The optical fibre channel is nonlinear, and the intensity-dependent Kerr nonlinearity limit has been suggested as a fundamental limit to optical fibre capacity. Current research is focused on whether this is the case, and on linear and nonlinear techniques, both optical and electronic, to understand, unlock and maximize the capacity of optical communications in the nonlinear regime. This paper describes some of them and discusses future prospects for success in the quest for capacity.

## 1. Introduction: optical fibre, the illusion of infinite capacity and why more bandwidth is needed

It is hard to imagine now that, since the introduction of the first optical fibre systems in the 1970s, pioneered in the UK’s historic and heroic Standard Telecommunication Laboratories and BT Research Labs (or the Post Office as it was then), the achievable transmission rates have increased by some six orders of magnitude, with three orders of magnitude increase in achievable distances. Optical fibre now underpins the global communications infrastructure with state-of-the art laboratory experiments achieving data rates in excess of 50 Tb s^−1^, using hundreds of wavelengths, over a single fibre core. The commercial systems lag these achievements but are not far behind. For many years it has been thought and claimed that, for all practical purposes, optical fibres have infinite capacity which would never be exhausted. And yet, the development of optical fibre technology has almost become the ‘victim’ of its own success.

For the last 40 years, the network providers have continually argued, with each generation, that optical fibre capacity of that generation is sufficient and little more is necessary. However, the availability of high-capacity fibre has provided the impetus for the growth of telecommunication, fuelling the development and proliferation of data services unfathomed and unfathomable at the time of the first systems being installed. The growth of the digital economy and the extension of our lives online provide a powerful argument that the provision of high capacity and ubiquitous fibre connectivity will lead to new services, and history has shown that it is a mistake to consider past or even current usage of scarce resources as a reliable guide to future demands in this fast growing and competitive domain. Indeed, a number of recent economics studies have analysed productivity increases through availability of broadband digital services and have demonstrated that these lead to additional increases in per annum productivity by as much as 0.4%, where total productivity growth in most economies is about 1.5%. Using the current UK gross domestic product of £1.7tr as an example, this would lead to an attributable gain of some £6.8bn [1].

It is hard to speculate about the future, but new services might include personalized genetic medicine for cancer or other diseases which requires transmission of DNA information with data requirements of some 1.5 GB per sequence. Treatments may require several sequences such that more than half a terabyte of data are transmitted, several times a day. Assisted ambient living and lifelong monitoring are also expected to result in significant data transmission demands, none of which can be satisfied with current capacity provisions. The ‘Internet of Things’, in which numerous consumer and sensor devices will also be connected to the Internet, has a projected number of 50 billion devices by 2020, and will add significant additional traffic to the network. Even without the addition of new services, current growth rates are greater than 20% per year with Cisco VNI forecasting [2] global public network traffic demands of some 130 exabytes (10^18^ bytes) per month in 2018—equivalent to 200 million DVDs. To put this in context—-500 exabytes is equivalent to the content of the Internet in 2009 (by 2013 it had reached 4000 exabytes!). Furthermore, the proliferation of data centres and cloud services has been accompanied by machine-to-machine traffic—not included in forecasts for traffic growth, but likely to add significantly to future capacity requirements.

**Figure 1.**
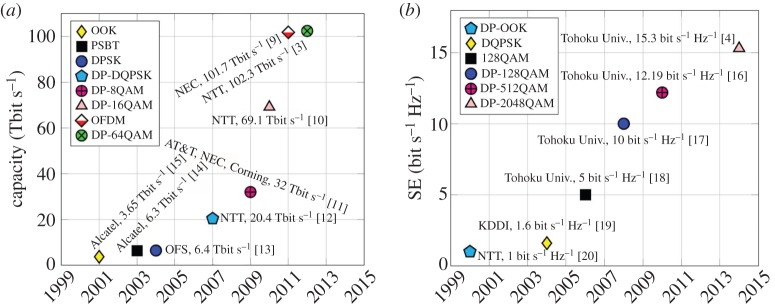
(*a*) Capacity and (*b*) SE growth demonstrated in state-of-the art laboratory and research experiments around the world. It should be noted that figures for SE are difficult to compare as experiments are performed over different bandwidths but the trend is interesting nonetheless [3,4,9–20].

So just how much data can be transmitted over optical fibre? Reported ‘record’ results vary by the metrics used to measure the capacity and distances over which transmission is reported. For example, the highest total capacity in bits per second, on a single fibre core is 102.3 Tbit s^−1^, transmitted over 240 km [3], using WDM DP-64QAM (wavelength division multiplexed dual-polarization 64-*ary* quadrature amplitude modulation) and digital coherent detection. Another metric is spectral efficiency (SE), measured in bit s^−1^ Hz^−1^ (or bit/s)/Hz and defined as the total capacity divided by the transmission bandwidth. The highest SE reported to date is 15.3 bit s^−1^ Hz^−1^ [4], corresponding to 66.6 Gbit s^−1^ single carrier DP-2048QAM over a relatively short distance of 150 km. Yet another measure is highest capacity–distance product over transoceanic distances. The current record for this metric is 52.2 Tbit s^−1^ over 10 230 km (534 Pbit s^−1^ km) and 54 Tbit s^−1^ over 9150 km (494 Pbit s^−1^ km) at spectral efficiencies of 5–6 bit s^−1^ Hz^−1^, using 180/200 Gbit s^−1^ DP-16QAM channels [5]. Often the SE is specified without reference to the bandwidth over which the transmission performance was investigated and, as we shall show later, it is much more challenging to achieve the same performance over a larger bandwidth. Yet another metric is the SE–distance product, with a record of 61 740 bit^−1^ Hz^−1^ km (6 bit s^−1^ Hz^−1^ over 10 290 km) [6], achieved using DP-16QAM channels. The highest capacity per channel is 10.2 Tbit s^−1^ (1.28 TBd) [7], achieved using optical time division multiplexing with DP-16QAM. Recent research on space-division multiplexing using multi-core fibre technology has yielded the highest total capacity per optical fibre [8] of 2.15 Pbit s^−1^ over 31 km of 22-core single-mode fibre. The relentless growth in experimentally achieved records is summarized in figure 1. It should be noted that, to make a fair comparison, only the useful information bits should be considered, i.e. the overheads added for forward error correction (FEC), necessary to correct the bit errors introduced in noisy and nonlinearly distorted signals, should be excluded. We shall return to this point in later sections but care should be taken with the analysis of reported results which often describe raw bits without subdividing them into bits carrying useful data and the coding overheads.

One simple solution to the capacity exhaust problem might be to install (or ‘light up’) additional fibres. The reluctance by operators to do this is a question of cost—adding more fibres, *N*, means that systems costs scale linearly with *N*, as they require *N* times more inline amplifiers, transceivers, power, and so on, resulting in a stagnating cost per bit transmitted. The growth in services and digital data traffic has been predicated on cost per bit going down which has now, within the current technology limits, asymptotically approached such low levels that further reductions appear challenging or even unrealizable. Something quite different must happen for cost per bit to continue on its path of reduction—such as dramatic capacity increases per system installed. This paper is intended to explain the needs and the challenges of increasing the amount of data which can be transmitted over optical fibres and the metrics used to measure them. The Kerr nonlinearity limit is explained, in the next section, together with a description of different techniques proposed to date to overcome it. One technique, namely multi-channel digital backpropagation (MC-DBP), is described in detail, from analytical (§3), numerical simulations (§4) and experimental perspectives (§5), over a range of modulation formats, and it is shown that it can lead to the doubling in the maximum transmission distance. The paper concludes on the prospects of future capacity increases, and discusses lower bounds as a function of fibre bandwidth, modulation formats and nonlinearity compensation, in §6.

## 2. Fibre capacity limits?

What limits the continued growth of achievable data rates in optical fibres? It was Claude Shannon who first introduced the concept of *channel capacity* in his 1948 paper [21]. The most famous result from this work says that for any channel, there exists a maximum transmission rate which can be achieved with an arbitrarily low error rate, and that above this quantity no reliable transmission is possible (see more details in §3). Unfortunately, for most (if not all) channels, no closed-form expression for the channel capacity exists. Fortunately, many channels (such as copper wires and wireless channels) can be accurately modelled using an additive white Gaussian noise (AWGN) channel model, in which case, the channel capacity is the elegant equation
2.1
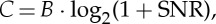
where *B* is the bandwidth and SNR is the signal-to-noise ratio of the channel.

Since its derivation, over 60 years ago, coding and information theorists have tried to approach this capacity limit with low-complexity coding schemes. For binary modulation, they have indeed succeeded, with performance within fractions of a dB [22]. A good review of these efforts can be found in [23].

The use of the information-theoretic capacity concept for the optical fibre channel dates back to the work of Splett *et al.* in 1993 [24]. However, it is only recently that this concept became popular in the optical fibre literature. This was prompted by the slowing in the dramatic capacity growth, outlined above [25], which had become constrained due to the fact that the optical fibre channel is *nonlinear*. The channel capacity analysis has turned out to be surprisingly difficult. A good review is given by Essiambre & Tkach [26].

The optical fibre channel is fundamentally nonlinear, which means that the refractive index of the propagation medium *n*_eff_ changes in response to the strength of the square of the electric field ***E*** or optical intensity *I*,
2.2
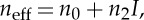
where *n*_0_ and *n*_2_ are the linear and nonlinear refractive indices, respectively; the normalized time averaged electric field ***E*** is related to optical intensity as 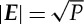, where *P* is the optical power in the fibre and *I*=*P*/*A*_eff_ with *A*_eff_ the effective area of the fibre. For silica, *n*_0_≈1.5 and 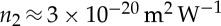. After a transmission distance *L*, the nonlinear optical Kerr effect will result in a phase shift, which, when added to the shift from linear propagation, results in a total phase shift, given by
2.3
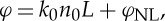
where *k*_0_=2*π*/*λ*_0_ is the free-space wavenumber, *λ*_0_ is the optical wavelength and *φ*_NL_ is a nonlinear phase shift. This phase shift depends on the optical signal power *P* as
2.4
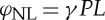
and *γ* is the fibre nonlinear coefficient, a measure of nonlinearity,
2.5
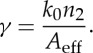
Transmission of more data within a finite bandwidth requires greater optical power levels *P*, which leads to the power-dependent nonlinear distortion of the transmitted data. This imposes a Kerr nonlinearity limit, sometimes referred to as the nonlinear Shannon limit. Strictly speaking, however, this limit is only a lower bound to the channel capacity, as it assumes that nonlinear interference is noise (whereas, as shown above, it is deterministic).

The propagation of the amplitude and phase of the electric field ***E*** is governed by the nonlinear Schrödinger equation (NLSE)
2.6
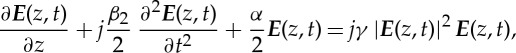
where *z* and *t* represent space and time, respectively. The NLSE in (2.6) captures the effects of Kerr nonlinearity (via the nonlinear parameter *γ*), loss (via the attenuation parameter *α*) and chromatic dispersion (via the group velocity dispersion *β*_2_) in the fibre. Furthermore, amplified spontaneous emission (ASE) noise is added by the erbium-doped fibre amplifiers (EDFAs) which interacts nonlinearly with the signals. Even in its simplest form (without attenuation and noise), the NLSE must be evaluated numerically. The algorithm typically used to this end is the split-step Fourier method, which iteratively and alternately solves the linear and nonlinear elements of the above equation. There are currently no closed-form expressions exactly describing fibre propagation for arbitrary input signal shapes.

Channel capacity calculations require a *channel model* (also known as channel law). The difficulty in quantifying the capacity of the nonlinear optical fibre channel is that no explicit (and simple) channel model exists. As mentioned above, the NLSE is a precise model; however, it is not explicit, i.e. the output of the channel is not expressed as a simple function of the input. The nonlinear fibre channel also introduces memory and the signal also interacts nonlinearly with the ASE noise. All these effects present a considerable challenge to the definition of the nonlinear channel model, a source of some debate of recent years, and a focus of much recent research activity, as explained by Agrell *et al*. [27].

There have been numerous proposals to overcome the Kerr nonlinearity limit or at least to mitigate the impact of nonlinearities. Some of the techniques are discussed in the following subsections.

### (a) Digital backpropagation

Digital backpropagation (DBP) is a fibre nonlinearity mitigation technique which can be applied both to a single channel and to a multi-channel (WDM) system. In the former case to compensate for self-phase modulation (SPM)—the nonlinear interference of a channel induced by its own electric field; in the latter case to mitigate for nonlinear distortions from both SPM and multi-channel nonlinearities (cross-phase modulation (XPM) and four-wave mixing (FWM)), caused by the electric field from adjacent WDM channels. This is done by simultaneously receiving and processing multiple optical channels using a high-bandwidth receiver.

In DBP, the NLSE in (2.6) is solved, in its inverse form, in the digital domain, by changing the sign of all the propagation parameters, allowing for the Kerr nonlinearity to be compensated, either fully or partially, i.e.
2.7
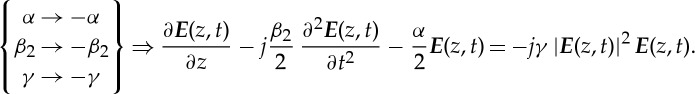
DBP aims at reconstructing the transmitted signal and is typically applied at the receiver. However, DBP can also be applied at the transmitter or split between the two. In all cases, however, complete reconstruction of the signal is not possible because of nonlinear mixing between the signal and the ASE noise as well as other stochastic effects such as polarization mode dispersion [28–30]. Applying DBP at the transmitter (sometimes termed as nonlinear pre-distortion or transmitter-based pre-distortion) or at the receiver gives approximately the same gains (determined by the accumulation of noise from the amplifier in the link) with one fewer amplifier noise contribution in the transmitter-based DBP case [31,32]. In the case of long transmission distances (greater than 10 spans), this difference is insignificant. In fact, it is clear that to reduce the contribution of noise, DBP should be split in the ratio of 50% : 50% between the transmitter and receiver. However, the greatest increase in the effectiveness of DBP can be achieved by reverse propagation of multiple channels, enabling the compensation of not only SPM but also inter-channel nonlinearities (XPM) [33]. The gains offered by DBP are discussed in detail in §§3, 4 and 5.

### (b) Optical phase conjugation

It is possible, using nonlinear optical processes, to exactly reverse the phase of a beam of light. The reversed beam is called a conjugate beam, and thus the technique is known as optical phase conjugation (also called spectral inversion). The role of the optical phase conjugator (OPC), typically positioned in the centre of a symmetrically amplified link, is played by a nonlinear element such as an optically pumped optical fibre. For best performance, a symmetric power profile is required, to ensure that distortion caused in the first half of the link is accurately reversed by that in the second half (see [34,35]). Recent achievements include dual-band optical phase conjugation of an optical superchannel using 75 km spans of standard single-mode fibre [35], where it was possible to substantially eliminate inter-channel nonlinear penalties; this nonlinearity compensation resulted in approximately 50% increase in transmission reach for six simultaneously transmitted 400 Gbit s^−1^ DP-16QAM superchannels, a record total bit rate of 2.4 Tbit s^−1^ using one dual-band OPC. In future, it may be possible to combine OPC with DBP for additional performance gains.

### (c) Nonlinearity-tailored detection

DBP is a zero-forcing equalization technique, and thus can be outperformed under certain circumstances by improved detection techniques that account for noise, nonlinearity and the memory of the channel. Given a noisy observation from the channel, optimal detection techniques minimize the error probability based on the noise distribution. For a multispan link, a closed form for this distribution is not available, and thus approximated solutions have been proposed [36]. Additionally, unlike the single span case, the interaction between signal and ASE noise limits the SNR at the receiver. As a result of this, the uncoded bit-error rate (BER) does not monotonically decrease as a function of the transmit power, even with improved detection. The problem of optimal detection of signals transmitted through a nonlinear channel with memory is theoretically important as it represents a preliminary step in the design of optimal receivers for more complex channel models. Recent promising results have been reported showing optimum detection for the single span fibre channel with a near-optimum detector in the presence of both nonlinear distortions and memory [37]. Despite the significant nonlinearity at the high values of transmitted powers used, it was shown that uncoded BER can be made arbitrarily low in this regime when the detector is tailored to the nonlinear properties of the channel, effectively acting as a ‘nonlinear matched filter’. More work remains to be done in this promising area.

### (d) Nonlinear Fourier transform and eigenvalue communications

This approach is a transmission and signal processing technique that makes positive use of the nonlinear properties of fibre channels. The NLSE is a member of a unique class of *integrable* nonlinear equations that can be solved via the so-called inverse scattering transform method [38]. This is an extension of the linear Fourier transform into nonlinear systems and is known as the nonlinear Fourier transform (NFT) [39–41]. The principle of the NFT involves the transformation of the effects of both nonlinearity and dispersion to a simple phase rotation of continuous nonlinear spectral data that play the role of a Fourier spectrum for nonlinear problems. The pioneering work of Hasegawa & Nyu [42] used discrete eigenvalues to encode and transmit information, as these are unaffected by dispersion and nonlinearity. There is currently much focus on this approach as it allows one to make positive use of the nonlinear properties of the fibre channel, since in integrable nonlinear channels, there are nonlinear normal modes that also propagate without any resulting nonlinear cross-talk; effectively in a linear manner. The conversion from the space–time domain into the nonlinear spectral domain and back is performed through the forward NFT and inverse NFT, respectively.

Alternative approaches to increasing capacity include a range of techniques which are in different stages of development such as coding tailored to the nonlinear channel, multi-channel all-optical and electronic regenerators or noise squeezing, wideband optical amplifiers—capable of taking advantage of the entire low-loss fibre bandwidth of 400 nm (1200–1600 nm), approximately 50 THz, rather than the limit imposed by the EDFAs of approximately 40 nm—and the use of negative *n*_2_ metamaterials which would allow for all-optical nonlinearity compensation.

This paper illustrates the capacity and distance gains that can be achieved through Kerr nonlinearity compensation by only one of these techniques (MC-DBP) through simulations, analytical approximations and experiments.

## 3. Estimating fibre capacity

### (a) Channel capacity and mutual information

The channel capacity can be defined for a continuous-time input continuous-time output channel, which is modelled for example via the NLSE in (2.6). However, the channel capacity is more commonly defined between the *discrete-time* input and output constellation symbols, i.e. when certain parts in the transmitter and receiver have been fixed (e.g. filtering, up/down-conversion, modulators, digital-to-analogue and analogue-to-digital convertors (ADCs)). For a detailed comparison between these two cases, see the paper by Agrell *et al*. [27].

The relationship between the input and output symbols is random, and thus it is modelled using a conditional probability density function (PDF) *p*_***Y***|***X***_(***y***|***x***), where ***X*** and ***Y*** are random vectors, both of length *n*, representing the transmitted and received symbols. The channel capacity is then defined as
3.1
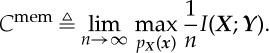
In (3.1), the optimization is over all input distributions *p*_***X***_(***x***) that satisfy a power constraint and *I*(***X***; ***Y***) is the mutual information (MI) between the random vectors ***X*** and ***Y***. MI describes the information that ***X*** and ***Y*** share. MI (measured in bits or bits s^−1^ Hz^−1^—by analogy with SE) is the reduction of uncertainty in ***X*** that we get from the knowledge of ***Y*** and vice versa. The channel capacity definition in (3.1) is general in the sense that it considers possible memory in the channel, and thus random vectors are considered. On the other hand, the expression in (3.1) is mathematically hard to analyse. Nevertheless, this expression was used to evaluate (numerically) the capacity of a heuristic channel model for dispersive fibre-optic links with finite memory in [43].

If the channel memory is discarded and the transmitted symbols are independent and identically distributed according to *p*_*X*_(x), a lower bound on the capacity can be found. In particular, if an *average* PDF *p*_*Y*_|_*X*_(*y*|*x*) is considered, we have
3.2
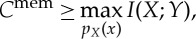
where the MI is defined as
3.3
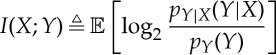
and 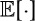 is the expectation operator.

If no maximization is performed in (3.3), yet another lower bound is obtained. This is the case when the constellation symbols are drawn with the same probability from a set of discrete constellation points (such as 16QAM or 64QAM), as usually done in practice. Mathematically, if we denote the transmitted QAM symbols by *X*^qam^, we obtain the following inequalities:
3.4
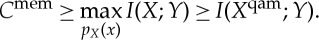
The expression in (3.4) simply shows that when the MI between the transmitted and received symbols *I*(*X*^qam^; *Y*) is considered, only a lower bound on the capacity is obtained.

### (b) Calculating the signal-to-noise ratio in nonlinear fibres

As discussed above, the performance of a long-haul dispersion-uncompensated transmission system is limited by the ASE noise from EDFAs and the Kerr nonlinearity. Near exact behaviour of optical data signals in dispersive and nonlinear fibre can be obtained from numerically solving the NLSE. However, as this is relatively time-consuming, it has been proposed that the nonlinear distortion in dispersion-uncompensated optical fibre transmission systems can be approximated as additive noise with a zero-mean Gaussian distribution, statistically independent from ASE noise. Whether this approximation represents a valid channel model in the high nonlinearity regime is still a much-debated open question, as it is based on the perturbation model approach to solving the NLSE (or other simplifying approximations). This so-called Gaussian noise model of nonlinear interference (*GN-model*), also predicts that the SNR tends to zero at high power levels which gives the incorrect impression of a channel capacity equal to zero in the highly nonlinear regime. In view of (3.4), however, this only means that a *lower bound* given by the GN-model tends to zero, and not the channel capacity. Nevertheless, the GN-model is a convenient approximation and, if used carefully, can provide valuable insights. In particular, the SNR given by the GN-model can be used to evaluate the *Q*^2^ factor or the MI in (3.3).

According to the GN-model, and under the assumption of incoherent accumulation of nonlinearity, the SNR in an optical communication system can be expressed as [28,44]
3.5
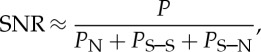
where *P* is the optical signal power, *P*_N_ is the total ASE noise in the transmission system, *P*_*S*–S_ is the total nonlinear signal–signal interaction, *P*_S–N_ is the total nonlinear signal–noise interaction and we have
3.6
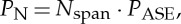

3.7
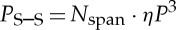

3.8
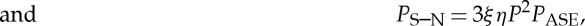
where *P*_ASE_ is the ASE noise power from one EDFA, *N*_span_ is the number of fibre spans, *η* is the nonlinear distortion coefficient according to the GN model and *ξ*=*N*_span_(*N*_span_−1)/2 is a factor depending on the number of spans. The ASE noise in the EDFA can be described as
3.9
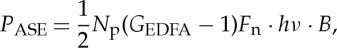
where *N*_p_ is the number of polarization states, *G*_EDFA_ is the gain of the EDFA, *F*_n_ is the EDFA noise figure, *h* is Planck’s constant, *ν* is the central frequency of the optical carrier and *B* represents the optical bandwidth.

For dual-polarization EDFA amplification, and Nyquist-spaced WDM transmission systems, the nonlinear distortion coefficient *η* can be approximated using the following expression [44]:
3.10
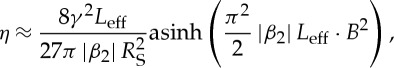
where *L*_eff_ is the fibre effective length and *R*_S_ is the symbol rate of the signal.

It should be noted that a source of debate relates to the form of the SNR expression in (3.5), where the noise is power dependent and the signal and noise cannot be separated, nor fully mitigated! This means that the SNR changes its physical meaning with power which has motivated researchers to seek a more accurate channel model to describe the nonlinear channel. This remains an active and dynamic research area.

### (c) Capacity lower bound calculations

The channel capacity of the nonlinear optical fibre channel (see *C*^mem^ in §3a) can be lower bounded using the expression for channel capacity for the memoryless AWGN channel in equation (2.1):
3.11
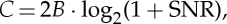
where the factor 2 introduced here describes two information-carrying polarization states considered in the fibre.

For a linear AWGN channel, the SNR is calculated from (3.5) and (3.6) as
3.12
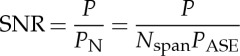
and the resulting lower bound in (3.11) becomes the actual channel capacity.

In the case of the digital signal processing (DSP) following coherent detection performing electronic dispersion compensation (EDC) only, nonlinear signal–signal interaction (much larger than signal–noise interaction in equation (3.5), which can therefore be neglected in this case) is included in the noise term, in addition to ASE, so that the SNR is calculated as
3.13
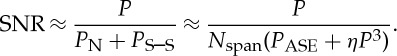
In the case of full-bandwidth DBP being applied as part of the receiver- or transmitter-based DSP, all nonlinear signal–signal interactions (including intra- and inter-channel nonlinearities) are removed, and only the signal–noise nonlinear interference is included in the noise term (in addition to ASE). The SNR is calculated using equation (3.5), as
3.14
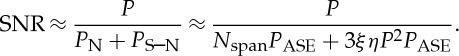
These expressions are applied to a variety of system simulations and experiments to test their validity in §4 and for capacity lower bound estimates in §6.

## 4. Numerical simulations of wavelength division multiplexing superchannel DP-64QAM transmission

As previously mentioned, in this paper, we focus on one of the many possible techniques to overcome the Kerr nonlinearity limit, specifically that of MC-DBP, illustrating its performance through numerical simulations, experiments and capacity estimates. In this section, numerical simulations of this technique are described. These are of a Nyquist-spaced 9-channel 32 Gbaud DP-64QAM WDM superchannel system, with a total raw data rate of 3.456 Tbit s^−1^. MC-DBP with 64QAM [45] is an advance on the previously reported results [33] showing doubling in achievable transmission distance. The achievable transmission distance of this superchannel system is evaluated both numerically, using the split-step Fourier algorithm, and analytically, using the perturbation-based GN-model, described in §3.

### (a) Simulation set-up

**Figure 2.**
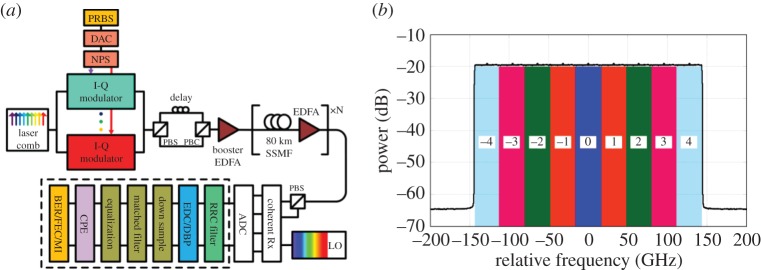
(*a*) Schematic of 9-channel DP-64QAM superchannel transmission system using the EDC or MC-DBP and (*b*) simulated transmission spectrum and the schematic of backpropagated bandwidth in the 9-channel DP-64QAM superchannel transmission system, where the frequency 0 Hz refers to the central frequency of the optical carrier (wavelength of 1550 nm). (PBS, polarization beam splitter; PBC, polarization beam combiner; NPS, Nyquist pulse shaping).

The set-up of the 9-channel 32 Gbaud per subchannel DP-64QAM superchannel transmission system is schematically illustrated in figure 2, and all numerical simulations were carried out based on the split-step Fourier algorithm solving the NLSE with a digital resolution of 32 Sa/symbol. In the transmitter, an optical frequency comb generator was applied to generate the nine phase-locked 32 GHz-spaced optical carriers for the subchannels (centred at 1550 nm). Digital-to-analogue convertors (DACs) with a resolution of 16 bit (to ensure no back-to-back implementation penalty) and root-raised-cosine (RRC) filters with a roll-off of 0.1% were used for the Nyquist-pulse shaping. The transmitted symbol sequences were decorrelated with a delay of 256 symbols using a cyclical time shift to emulate the independent data transmission in each subchannel and the sequences in each polarization were also decorrelated with a delay of half the sequence length. The standard single-mode fibre (SSMF) was simulated based on the split-step Fourier method with a step size of 0.1 km, and the detailed system parameters were: span length of 80 km, *α*=0.2 dB km^−1^, 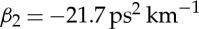 and *γ*=1.2 W^−1^ km^−1^. Negligible fibre PMD was assumed. The noise figure of the EDFA was set to *F*_n_=4.5 dB. At the receiver, the signal was mixed with a free-running local oscillator (LO) laser using an ideal 90^°^ optical hybrid and sampled at 32 Sa/symbol, without any bandwidth limitation, which allowed an ideal and synchronous detection of all the in-phase and quadrature signal components over the whole superchannel bandwidth. The DSP modules included a RRC filter for selecting the MC-DBP bandwidth, followed by MC-DBP (or linear EDC), down-sampling (to 2 Sa/symbol), matched filtering, multiple modulus algorithm equalization, ideal carrier phase estimation (CPE), symbol de-mapping and BER estimation. The simulated spectrum of the 9-channel DP-64QAM coherent transmission system is shown in figure 2*b*, where the number represents the subchannel index within the entire superchannel.

The EDC was realized using a frequency domain equalizer [46], and MC-DBP algorithm was implemented using the reverse split-step Fourier method applied to the signal nonlinear propagation based on the Manakov equation (2.6) with a 0.5 split ratio for the chromatic dispersion compensation [30,47]. An ideal RRC filter was applied to select the desired backpropagated bandwidth for the MC-DBP, and also to reject the unwanted out-of-band ASE noise. The backpropagated bandwidth was 32 GHz for the single-channel DBP, increasing to 288 GHz for 9-channel (full-bandwidth) DBP. The sampling rate was 32 Sa/symbol both in the EDC and the MC-DBP operations. A matched filter was used to select the subchannel of interest and to optimize the SNR of the processed signal. The multiple modulus algorithm with 21 taps was used for the polarization equalization and residual linear impairment equalization. The CPE was realized using ideal phase estimation, assuming there is no phase noise in the Tx and LO laser. Finally, symbol de-mapping and BER estimation were carried out to assess the transmission performance of the measured subchannel based on 2^18^ bits, with a pseudo-random bit sequence pattern length of 2^15^−1.

### (b) Performance of multi-channel digital backpropagation in superchannel transmission

**Figure 3.**
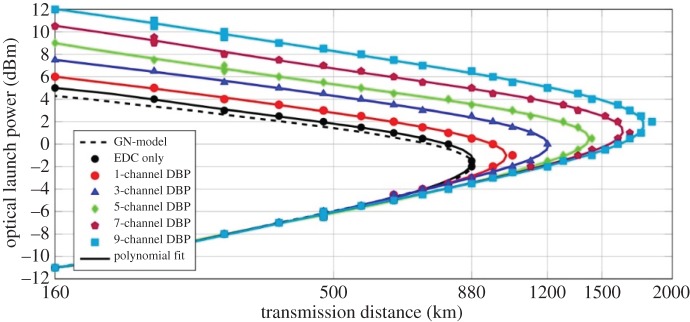
Maximum transmission distance as a function of different optical launch powers at a pre-FEC BER of 1.5×10^−2^ in the 9-channel DP-64QAM superchannel transmission system using EDC and MC-DBP. The markers are the simulation data, and the solid line is the 5th order polynomial fit. The approximation obtained used the GN-model is also shown for EDC (dashed line).

In all simulations, the MC-DBP algorithm was run with 800 steps per span, with a nonlinear coefficient of 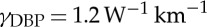. The performance of the transmission system with the MC-DBP algorithm applied at the receiver was investigated for the case in which the linewidths of both the transmitter and the LO lasers were set to 0 Hz to remove the influence from phase noise. The achievable transmission distance for different launch powers for the central subchannel (with channel index 0 in figure 2*b*) in the 9-channel DP-64QAM Nyquist transmission system was calculated and is shown in figure 3. The BER threshold was set to 1.5×10^−2^ (*Q*^2^ factor of approx. 6.7 dB), corresponding to a 20% overhead hard-decision FEC threshold, which is assumed to correct the BER to below 10^−15^ [48]. These results were obtained using either EDC only or MC-DBP with different backpropagated bandwidths. From the results in figure 3, it can be seen that when EDC only is applied, the maximum transmission distance which can be achieved is 880 km (11 SSMF spans) at the optimum launch power (−2 dBm). Single-channel DBP can give an improvement of approximately 18% in the transmission distance (1040 km at launch power of −1 dBm), whilst when all 9 channels are simultaneously detected and the entire spectrum is digitally backpropagated to undo the nonlinearities, an improvement of over 109% in the transmission distance (1840 km at a launch power of 2 dBm) is obtained. The improved performance in the achievable superchannel transmission system can be seen with each increment in the MC-DBP bandwidth. In addition, the analytical prediction of the transmission performance was also carried out using the perturbation theory-based GN model (of §3) [44,49], with good agreement between the analytical prediction and the simulated reach curve with EDC.

**Figure 4.**
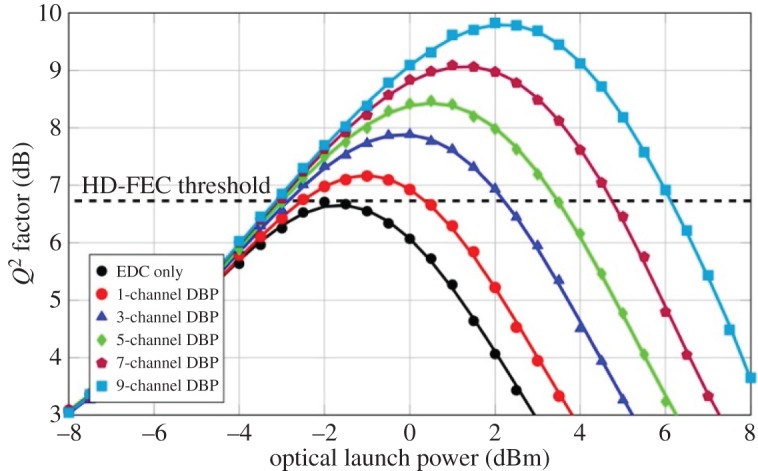
*Q*^2^ factor at 880 km (11 SSMF spans) in the 9-channel DP-64QAM transmission system using the MC-DBP over different backpropagated bandwidths.

The performance of the MC-DBP in the Nyquist-spaced 9-channel DP-64QAM superchannel transmission system was also investigated in terms of *Q*^2^ factor for different optical launch powers, as shown in figure 4. The transmission distance considered was the maximum achievable distance—880 km (11 SSMF spans)—for the system using EDC only. All the *Q*^2^ factors are converted directly from the measured BER values. It can be seen from figure 4 that the performance of the MC-DBP in the superchannel transmission system improves with the increment of the backpropagated bandwidth. The best achievable *Q*^2^ factor with EDC only is approximately 6.7 dB at an optimum launch power of −2 dBm. When the single-channel DBP is employed for SPM compensation, the best achievable *Q*^2^ factor was improved to approximately 7.2 dB at an optimum launch power of −1 dBm. When the 9-channel (full-bandwidth) DBP is applied over the whole superchannel for compensating the SPM, the XPM and the FWM simultaneously, the best achievable *Q*^2^ factor is improved to approximately 9.8 dB at the optimum launch power of 2 dBm. The *Q*^2^ factor gain of the 9-channel (full-bandwidth) DBP is approximately 3.1 dB compared with the EDC only case, and is approximately 2.6 dB compared with conventional single-channel DBP case. This investigation on the operation and the optimization of the MC-DBP indicates the optimal operation of the full-bandwidth DBP and a benchmark for the evaluation of the 9-channel DP-64QAM Nyquist superchannel transmission system.

### (c) Digital backpropagation optimization and complexity

Signal equalizations in both cases of EDC and full-bandwidth DBP were applied over the entire superchannel bandwidth in all the above analysis. However, it is a requirement that the dispersion compensation in the MC-DBP (or full-bandwidth DBP) be applied simultaneously over all backpropagated subchannels, since all the information of the nonlinear interference in the involved subchannels is required for the MC-DBP to cancel both the intra-channel and the inter-channel nonlinearities. For EDC, it is only a requirement to apply the filtering over the bandwidth of the channel itself.

All the above numerical simulations have been implemented for a 9-channel 32 Gbaud Nyquist-spaced DP-64QAM optical transmission system. The findings can also be qualitatively applied to transmission systems using different modulation formats and different numbers of WDM subchannels. In addition to MC-DBP, as noted in the introduction, mid-span OPC is also a promising approach to compensate the fibre nonlinearities over multi-channel WDM transmission systems, where the phase conjugation of the transmitted signal is employed to generate, over the course of the second half of the transmission link, an opposite nonlinear phase shift for compensating the nonlinear effects generated in the first half of the transmission link [50,51].

**Figure 5.**
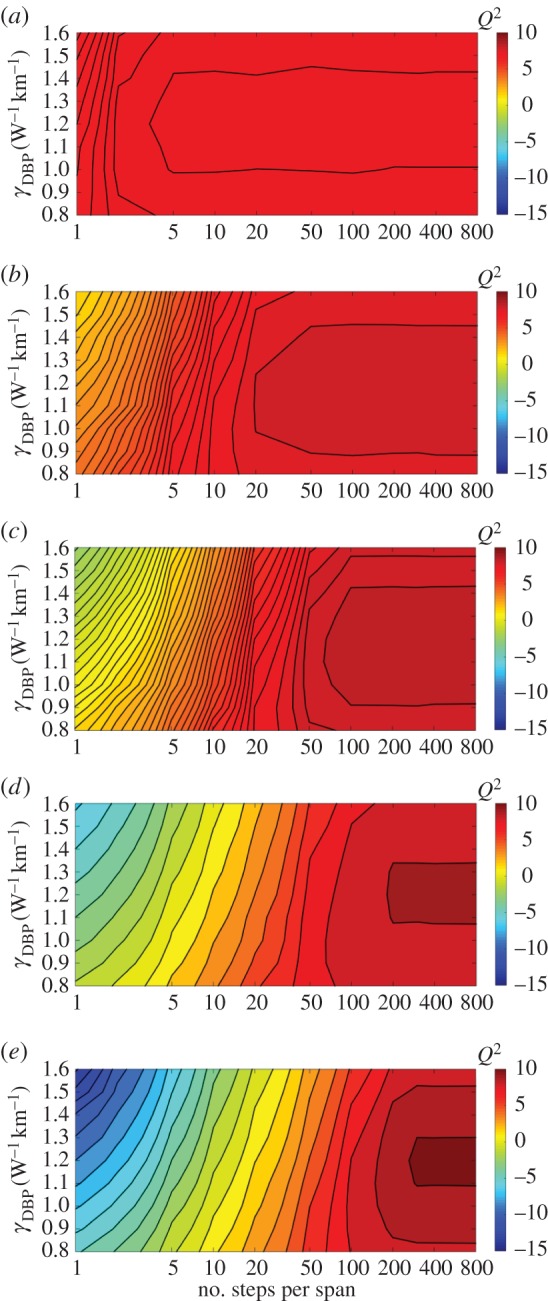
Optimization of MC-DBP for different numbers of backpropagated subchannels: *Q*^2^ factor as a function of the nonlinear coefficient and the number of steps per span used in the DBP algorithm. (*a*) 1-channel DBP, (*b*) 3-channel DBP, (*c*) 5-channel DBP, (*d*) 7-channel DBP and (*e*) 9-channel DBP.

It is interesting to explore the efficacy of the MC-DBP algorithm. For simplicity, the linewidths of both the transmitter and the LO lasers can be set to 0 Hz for this analysis. The operation of the MC-DBP can then be investigated for different backpropagated bandwidths in terms of the number of steps per fibre span and nonlinear coefficient parameter *γ*_DBP_. We illustrate this for the transmission distance of 880 km (11 SSMF spans), as before. The optimum launch power was always selected to achieve the lowest BER in the central subchannel for the particular MC-DBP bandwidth used, as shown in figure 5. It can be seen that for the nonlinear coefficient of *γ*_DBP_=1.2 W^−1^ km^−1^, in single-channel DBP (32 GHz), the minimum number of steps per span is approximately 4, increasing to 300 in 9-channel (full-bandwidth) DBP. It is found that a good agreement between the minimum number of steps per span and the quadratic polynomial fit can be achieved.

**Figure 6.**
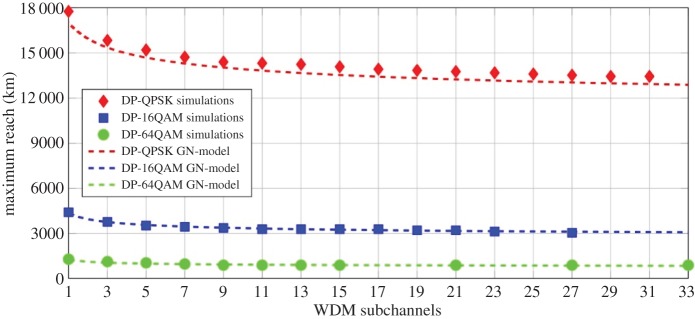
Maximum reach versus number of channels for different modulation formats (at a pre-FEC BER of 1.5×10^−2^). Comparison of results from numerical simulations and analytical calculations using the GN-model.

There has been much discussion about the complexity of MC-DBP, which is indeed very high in comparison with EDC [30]—possibly too high for it to become practical in the near future for large bandwidth systems. However, simulations of achievable transmission distance using both simulations and the GN-model (albeit at the optimum power, and thus not in a very highly nonlinear regime) show that, for a given channel spacing or SE, although achievable reach reduces with bandwidth, as shown in figure 6 (reach versus number of channels for different modulation formats) it reduces logarithmically with increasing number of transmitted channels (a parameter which is dependent on the modulation format). This quasi-saturation is promising for MC-DBP, as it means that, to a first approximation, only a limited subset of channels needs to be backpropagated to obtain the majority of the achievable benefit. In the example above, for the DP-64QAM case, only approximately 5 channels need to be backpropagated, despite a much greater number of channels transmitted. This small number of channels accounts for most of the nonlinear interaction and resultant distortion with channels further away contributing significantly less distortion. Digital back propagation is fast becoming a practical reality with first DSP chips currently being tested,^1^ and the significant progress made in this area is likely to continue.

## 5. Experimental transmission of Nyquist-spaced DP-64QAM superchannel and multi-channel digital backpropagation

Having investigated transmission by numerical simulations in the previous section, we now focus on experimental demonstrations of multi-channel transmission experiments and the effectiveness of practically implemented MC-DBP. MC-DBP was previously demonstrated experimentally using a spectrally sliced coherent receiver to achieve a *Q*^2^ factor gain of approximately 1 dB by backpropagating a 5-channel 30 GBd DP-16QAM superchannel [52]. A single coherent super-receiver, which simultaneously receives and demodulates multiple optical subchannels, was demonstrated by Tanimura *et al.* [53] to backpropagate a 4-channel 28 GBd optical superchannel and an enhanced *Q*^2^ factor margin was demonstrated at a fixed distance of 1000 km. A single digital coherent receiver was also used to simultaneously receive a 7-subchannel 10 GBd subchannel Nyquist-spaced DP-16QAM superchannel and MC-DBP was subsequently employed to increase the maximum reach of the transmission system by 85%, from 3190 to 5890 km [33].

Seven subchannels were used in the experiment described in this section, as in [54] (rather than the nine considered in the simulations described in §4) so that the entire superchannel could be detected simultaneously within the super-receiver analogue electrical bandwidth of 63 GHz, as described below. The entire DP-64QAM signal with seven 10 GBd subchannels was simultaneously received using a digital coherent super-receiver and the performance of each subchannel was analysed after transmission over 1280 km of SSMF, both with and without MC-DBP. The role played by coding for adaptive FEC is also described.

### (a) Transmission experimental set-up

**Figure 7.**
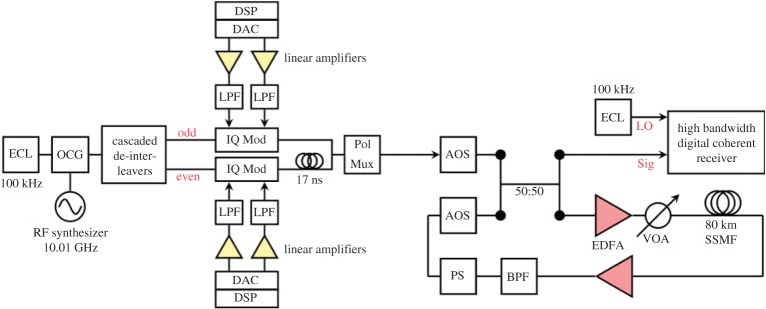
DP-64QAM superchannel experimental set-up. AOS, acousto-optic switch; PS, polarization scrambler; VOA, variable optical attenuator; BPF, band pass filter.

The 7-subchannel 10 GBd DP-64QAM superchannel transmission system is shown in figure 7. The output of a single laser source was passed through an optical comb generator, which was used to produce seven frequency and phase-locked comb lines, each separated by a channel spacing of 10.01 GHz. A slight offset in channel spacing (relative to the Nyquist frequency) of 10 MHz was required in order to avoid a previously observed artificial performance enhancement, which occurs when using bulk modulated odd and even subchannels spaced at the Nyquist frequency. The multi-level electrical drive signals used for the 64QAM format were generated offline and were digitally filtered using a RRC filter (roll-off factor: 0.1%) to constrain the optical bandwidth of each subchannel. The resulting in-phase (I) and quadrature (Q) signals were loaded onto a pair of DACs operating at 20 GSa/symbol (2 Sa/symbol). The odd and even subchannels were independently modulated using two complex IQ modulators, which were subsequently decorrelated before being combined and polarization multiplexed to form a Nyquist-spaced DP-64QAM superchannel. A conventional recirculating loop configuration was used for the emulation of long-haul transmission and included a single 80 km span of SSMF. Finally, the polarization diverse coherent receiver had an electrical bandwidth of 70 GHz and used a second 100 kHz external cavity laser as an LO for the coherent detection process. The emission frequency of the LO was set to coincide with the central subchannel of the DP-64QAM superchannel and the received signals were captured using a 160 GSa/symbol real-time sampling oscilloscope with 63 GHz analogue electrical bandwidth. The key receiver DSP blocks were identical to that illustrated in figure 2*a*.

Three figures of merit were employed to measure the performance of the superchannel transmission system. These include the BER calculated before FEC, the BER calculated after FEC and the MI. The combination of multi-level modulation, such as the 64QAM format used in this work, and FEC is known as coded modulation (CM). In a CM-based optical communications system, the transmitter passes information bits into a binary encoder, which adds redundancy that is used for error correction at the receiver and operates at a code rate *R*_SD_=*N*_b_/*N*_c_, where *N*_b_ is the number of information bits and *N*_c_ is the number of coded bits. The coded bits are mapped to a set of discrete constellation points (64 in this case) using a memoryless mapper and are subsequently transmitted over the optical channel. The receiver consists of a memoryless demapper and a FEC decoder. A soft decision FEC (SD-FEC) decoder was used in this experiment to correct bit errors at the receiver (described in detail in [33]), albeit for a reduction in SE due the added redundancy, which is scaled by the required code rate. The pre-FEC BER was calculated before the SD-FEC decoder by passing the received symbols through a hard decision (HD) de-mapper, while the post-FEC BER is calculated by comparing both the encoded and decoded bits, which are obtained at the output of the SD-FEC implementation [54] The MI between the transmitted and received symbols is a key quantity in information theory, as it represents the largest achievable information rate for any CM-based system. It is calculated between the transmitted and received symbols using Monte Carlo integration and is described in detail in the methods section of [34]. The MI is normalized by dividing by *m*, and thus provides the largest code rate, *R*, that can be used to achieve an arbitrarily low BER for a given CM system. For a practical FEC implementation, the total code rate of the error correcting scheme must be less than or equal to *R*. This code rate can be easily converted to a FEC percentage overhead through OH %=[(1/*R*)−1]×100.

In this experimental demonstration, a low-density parity-check-based SD-FEC scheme was implemented offline in Matlab and is again detailed in the methods section of [34]. An outer HD staircase code (SCC) that had a code rate *R*_HD_=16/17 was assumed. This outer HD-FEC code was chosen as it produces a BER of 10^−15^ for a post-FEC BER of 4.7×10^−3^. Therefore, if the post-FEC BER, measured at the output of the SD-FEC implementation, was below the threshold for the outer concatenated SCC, then a BER of 10^−15^ was assumed to have been achieved. The concatenated code rate *R*_SD_*R*_HD_ must be less than or equal to *R*, as calculated by normalizing the MI.

### (b) Pre- and post-forward error correction bit-error rate performance

**Figure 8.**
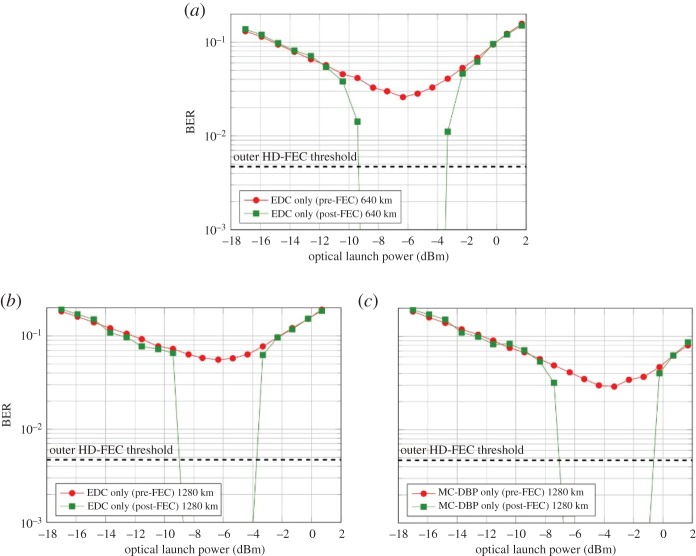
Pre- and post-FEC BER as functions of launch power for the central subchannel. (*a*) EDC only after 640 km of SSMF (*R*_SD_=5/6), (*b*) EDC only after 1280 km of SSMF (*R*_SD_=3/4) and (*c*) MC-DBP after 1280 km of SSMF (*R*_SD_=5/6).

The pre- and post-FEC BER as a function of launch power for the central subchannel is displayed in figure 8. After 640 km transmission over SSMF and with EDC only, the pre-FEC BER reduced linearly as the launch power increased from −18 dBm to −8 dBm, as seen in figure 8*a*. The minimum BER was achieved at an optimum launch power of −6.5 dBm, after which the pre-FEC BER began to increase with higher launch power due to signal distortions arising from fibre nonlinearity. The corresponding post-FEC BER, measured at the output of the SD-FEC decoder with *R*_SD_=5/6 (corresponding to an overhead (OH) of 20%), is also shown in figure 8*a*. This reduced the BER below the threshold for the outer HD SCC and also provided a launch power margin of approximately 6 dB (at the HD-FEC threshold). When the transmission distance was increased to 1280 km (figure 8*b*), the pre-FEC BER at the optimum launch power increased from 2.6×10^−2^ to 5.6×10^−2^. Therefore, a SD-FEC code with *R*_SD_=3/4 (33.33% OH) was required to maintain a consistent launch power margin at the HD-FEC threshold. However, when MC-DBP was employed in the receiver DSP, the optimum launch power increased by 3 dB to −3.5 dBm and there was a corresponding decrease in the pre-FEC BER to 2.9×10^−2^, as shown in figure 8*c*. This enabled a reduction in the required OH for the SD-FEC decoder to 20%, which was identical to that used for the EDC only case after 640 km transmission. Therefore, this resulted in the same SE of 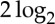
(64) *R*_SD_ 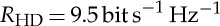 for the central subchannel but, significantly, at double the transmission distance. If the aim is to maintain the SE over a given distance or, perhaps even more importantly from the overall capacity point of view, over a much wider bandwidth, then the application of a judiciously chosen combination of code and coding overhead (or equivalent code rate) can help achieve this.

### (c) Mutual information as a function of transmission distance

**Figure 9.**
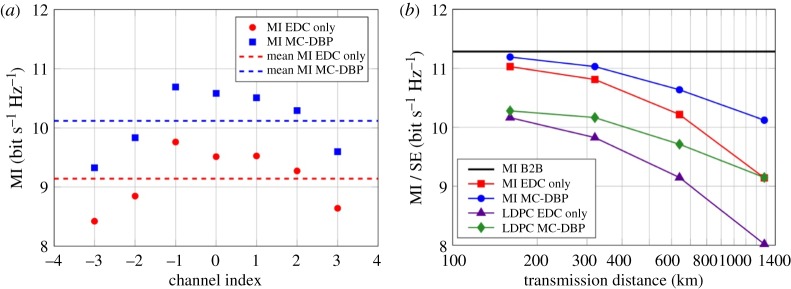
(*a*) MI for each subchannel with and without MC-DBP after transmission over 1280 km of SSMF. (*b*) Mean MI and SE of the 7-subchannel DP-64QAM signal.

In order to evaluate the maximum performance for this given optical communications system, the MI, as defined in §3a, was calculated as a function of transmission distance and for all 7 subchannels. Figure 9*a* illustrates the MI for all 7 subchannels of the DP-64QAM signal after transmission over 1280 km of SSMF. When only EDC was employed in the receiver DSP, a maximum MI of approximately 9.8 bit s^−1^ Hz^−1^ was achieved per subchannel. The MI varied by approximately 0.2 bit s^−1^ Hz^−1^ over the central 3 subchannels, but reduced significantly towards the edge subchannels. This deterioration in performance is attributed to the frequency-dependent effective number of bits in the receiver ADCs and is inherent to wide bandwidth receivers that simultaneously capture and process more than one optical channel. In order to achieve error free transmission, the code rate of the FEC implementation needs to be tailored for each subchannel, depending on the SNR after coherent detection. This will ensure that the number of information bits transmitted over the channel is maximized. MC-DBP increased the MI of each subchannel by 1 bit s^−1^ Hz^−1^ and, thus, yielded a mean MI for the DP-64QAM superchannel of 10.1 bit s^−1^ Hz^−1^.

The corresponding average MI for all 7 subchannels, as a function of transmission distance, is displayed in figure 9*b*. The B2B mean MI of the DP-64QAM superchannel was 11.3 bit s^−1^ Hz^−1^ and provided the maximum achievable information rate of the system. After transmission over 160 km of SSMF and with EDC only (circles), the MI reduced to 11 bit s^−1^ Hz^−1^, which decreased further to 9.15 bit s^−1^ Hz^−1^ at the maximum transmission distance of 1280 km. When MC-DBP was also used in the receiver DSP (squares), the MI increased for all recorded transmission distances. A marginal improvement in MI was achieved at a transmission distance of 160 km; however, at the maximum reach, there was a 1 bit s^−1^ Hz^−1^ increase in the MI relative to the EDC only case. The mean SE of the DP-64QAM superchannel system was calculated by tailoring the code rate of the SD-FEC implementation for each subchannel in order to achieve a post-FEC BER below 4.7×10^−3^, which corresponds to the threshold of the outer SCC. This was performed for each transmission distance and is also displayed in figure 9*b*. A constant 1 bit s^−1^ Hz^−1^ penalty in the SE was incurred using the SD-FEC decoder at all transmission distances. For EDC only (triangles), the achieved SE followed the same trend as the estimated MI, reducing from 10.16 bit s^−1^ Hz^−1^ at a transmission distance of 160 km to 8 bit s^−1^ Hz^−1^ at 1280 km. Again, MC-DBP (diamonds) provided a gain in SE of 1 bit s^−1^ Hz^−1^ at the maximum reach. However, for a fixed mean SE of 9.15 bit s^−1^ Hz^−1^, EDC only achieved a transmission distance of 640 km, while the reach was extended to 1280 km when MC-DBP was employed in the receiver DSP. This represents a 100% reach enhancement due to MC-DBP and is in excellent agreement with the central subchannel performance shown in figure 3.

## 6. Just how much can capacity be increased?

Clearly, a number of techniques can be effective in overcoming what has previously been assumed to be the Kerr nonlinearity limit, although the greatest effectiveness of the aforementioned techniques appears to be in increasing the transmission distance, where the capacity and SE can be maintained over as much as double the distances compared with a linearly compensated system. What about overall increases in the maximum fibre capacity?

Increasing the overall capacity is a major scientific challenge. Although for an unrepeated link, one which does not contain amplifiers, the capacity can be defined by the quantum limit of detection (i.e. shot noise [55]), recent publications [56,57] asserted that for any amplified link, an upper bound on the capacity, *C*^*mem*^ in (3.2), is given by the log_2_(1+*SNR*) expression for an equivalent AWGN channel where the SNR is calculated using only the ASE noise. This result means that the capacity of the optical fibre channel is not above a log_2_(1+SNR) expression, and that dispersion and nonlinearity do not increase capacity. The implication of this result is that the best we can hope for is to ideally compensate Kerr nonlinearities and dispersion, resulting in a linear AWGN channel and under this assumption the nature of logarithmic function means that increasing power in the fibre will lead to progressively smaller increases in channel capacity.

As already mentioned, the channel capacity of a communication channel is the maximum rate at which information can be transmitted with an arbitrarily low error probability. Indeed, the recent vociferous scientific debates about the channel capacity of different channels have led to the realization that all the optical channels discussed are indeed different: they depend on fibre and amplifier type, span length and the in-span compensation scheme—such as optical phase conjugation or optical regeneration. In the case of the nonlinear channel, the channel properties are power dependent at the onset of significant nonlinearity or in the high power regime. Each of these different channels will have its own channel capacity and comparisons must be made with utmost care.

Assuming an AWGN channel one could use the GN-model to estimate the required optical power to ensure approximately a factor of 10 increase in capacity compared to the current record. Using the approximate expressions from §3, it is possible to explore the limits of what is possible through nonlinearity compensation. Using somewhat unrealistic, grossly simplifying, assumptions (e.g. wavelength-independent dispersion and loss coefficients), it is possible to explore what capacity gains might be achievable through (i) complete nonlinearity compensation and (ii) an increase in usable fibre bandwidth beyond the current EDFA bandwidth limit of approximately 35 nm (4.3 THz) to 50 THz (i.e. the full standard single-mode fibre bandwidth), approximately an order of magnitude increase. The parameters which have been assumed for the calculations include: 2 polarizations, 50 THz bandwidth (50 Gbaud ⋅ 1000 channels), C-band (4.3 THz, 50 Gbaud ⋅ 86 channels), lumped amplification with *F*_n_=3 dB noise figure, group velocity dispersion *β*_2_=−21.7 ps^2^ km^−1^, zero PMD, nonlinear coefficient *γ*=1.2 W^−1^ km^−1^, attenuation *α*=0.2 dB km^−1^ and 80 km per span.

**Figure 10.**
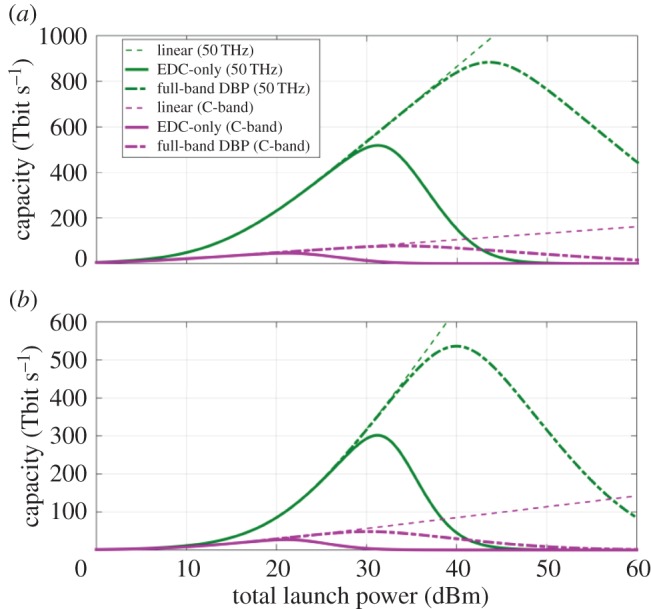
Capacity versus total launch power for C-band and 50 THz at (*a*) 2000 km (25 spans of 80 km) and (*b*) 10 000 km (125 spans of 80 km).

The obtained results are illustrated in figure 10, where capacity is plotted against power for two different bandwidths and distances (2000 km—a long-haul terrestrial link; and 10 000 km—equivalent to a subsea, transoceanic system). It can be seen from figure 10 that, for an SE of approximately 10 bit s^−1^ Hz^−1^ per polarization, it would be possible to transmit 1 Pbit s^−1^ in the linear regime with quantum noise-limited amplifiers at the launch power of 45 dBm (approximately 30 W total launch power). This would exceed the current record for maximum system capacity of 100 Tbit s^−1^ by approximately a factor of 10. Taking nonlinearity of the channel into account leads to a reduction in capacity of approximately 500 Tbit s^−1^. Much of this capacity can be recovered by full field DBP, which increases capacity to 850 Tbit s^−1^. Without relying on the use of spatial modes, a factor of greater than 10 is difficult to envisage at present because of the difficulty of increasing transmitted capacity by purely increasing the cardinality of modulation format. In the examples given in this paper—DP-64QAM requires 2^6^ distinct amplitude-phase levels per polarization, yielding 12 bit s^−1^ Hz^−1^ maximum SE (or 6 bit s^−1^ Hz^−1^ per polarization). These simple calculations allow us to explore the price one would have to pay in terms of SNR required to achieve these gains, and the associated optical power. Note, again, that there is an assumption of the fundamental limit in amplifier noise of 3 dB.

**Figure 11.**
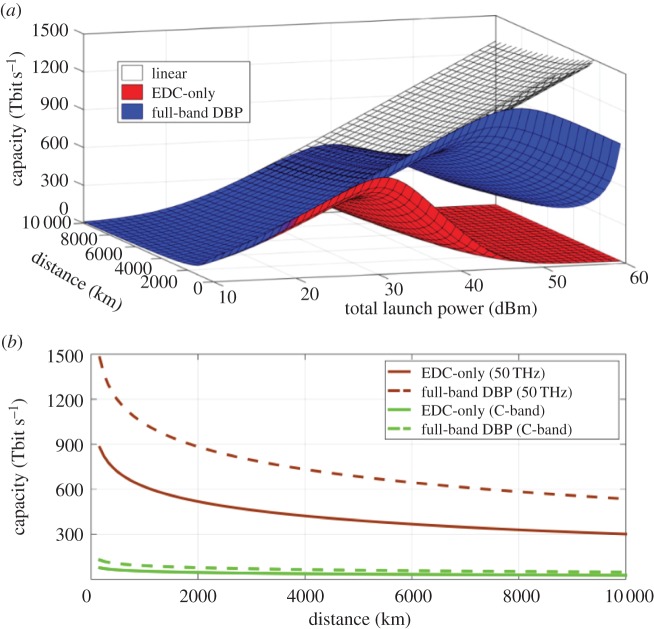
(*a*) Capacity versus transmission distance and total launch power at 50 THz bandwidth and (*b*) capacity versus distance for optimum launch power for both 50 THz bandwidth and C-band.

Using the equations from §3b, a three-dimensional sweep of signal launch power, capacity and transmission distance is plotted in figure 11*a*, showing how these parameters are related. Figure 11*b* shows the capacity obtained when the launch power is optimized for each distance and both the EDC only and full-band DBP. For the EDC only case, the optimum launch power is constant with respect to distance and given by 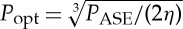. For the full-bandwidth DBP case, the optimum launch power depends on transmission distance (number of spans), and then we have 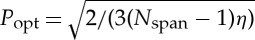 with *N*_span_≥2.

Considering the results in figure 11*b*, it is possible to use empirical data to estimate the feasibility of achieving, or indeed of going beyond, the above scenario. To encode 850 Tbit s^−1^ in 50 THz optical bandwidth requires 8.5 bit/symbol/polarization at the Nyquist limit. To date, the record achieved SE for gigabit-class fibre-optic communications is 7.65 bit/symbol/polarization (considering FEC overhead, which can be as high as 100%, depending on the combination of nonlinearity, transmitted format and distance) [4], encoded using DP-2048QAM. Note that, although this does not achieve the required information per symbol, higher order constellations could bridge the gap in this case. The limiting factors in increasing the order of QAM beyond this level include: the availability of high-speed, high-resolution DACs and ADCs, laser linewidth and the accuracy of the DSP implementation. All of these factors will in some way limit the achievable SNR; however, current hardware limitations are unlikely to persist, meaning that the above scenario may, at some point, be experimentally demonstrated.

To explore the possibilities beyond the 850 Tbit s^−1^ transmission considered above, it is instructive to look at (traditional) coaxial communications, where high-order QAM signal generation and detection is more advanced. The current *Data Over Cable Service Interface Specification (DOCSIS) 3.1* standard documents the required capability of transmitting 4096QAM, and the possibility of transmitting 16384QAM [58], which encodes 14 bit/symbol. On the horizon, there is the possibility of transmitting 65536QAM within DOCSIS; however the SNR requirements for this format are in excess of 60 dB. Considering, again, the 50 THz bandwidth used in the above transmission scenario, but assuming that the SNR is, now, sufficient to transmit DP-65536QAM, the maximum capacity would be limited to 1.6 Pbit s^−1^, which, in spite of the staggering SNR requirement, is less than a factor of two beyond what could be achieved using the already-demonstrated DP-2048QAM. Thus, for future ultra-high-capacity systems, there is a need to unlock ever wider optical fibre bandwidth for transmission. This would include, for example, development of new ultra-wideband amplifiers, sources, detectors and fibre designs combined with space-division multiplexing. The latter may include multiple fibres, multiple cores within a single fibre or multiple modes within a single fibre. Metamaterials could offer all-optical nonlinearity compensation through the use of negative *n*_2_ materials [59]. There remain many challenges to overcome the nonlinear limits of optical fibre communications, to show that already realizable capacities can be achieved over ever-greater distances. Finally and optimistically, even relatively small increases in the achievable transmission rates may be converted into large gains in overall *network capacity* for networks operated in the nonlinear regime. This is a relatively unexplored area and requires the evaluation of overall network throughput, taking channel properties into account. Given that the definition of network throughput and/or capacity is itself under debate—how much more so techniques for quantifying it!

In both channel and network modelling, some of the controversial challenges relate to the calculation of channel capacity (as was highlighted in the course of the discussion meeting) and the difficulties of common definitions. This may be because, to quote Caves & Drummond [60], ‘A communication theorist defines a channel by specifying input and output alphabets and by giving conditional probabilities that characterize channel noise; he or she then puts primary emphasis on deriving rigorous mathematical consequences … whether or not the channel has a physical realization’, whereas, ‘A physicist … puts primary emphasis on a physical realization, because a physicist is interested in how physical law affects the performance of communication systems.’ The difference between them ‘can make the capacity go to zero when the two groups try to communicate.’ The vital importance of the Royal Society discussion meeting was to bring these groups together. Only by speaking a common language can communication and information theorists work with physicists and engineers to overcome the grand challenges of maximizing the capacity of optical communications.

## Footnotes

1 see http://www.ntt-electronics.com/en/news/2015/9/200g-high-performance-dsp.html for a recent press release on the use of DBP.
